# Isocitrate Dehydrogenase of *Helicobacter pylori* Potentially Induces Humoral Immune Response in Subjects with Peptic Ulcer Disease and Gastritis

**DOI:** 10.1371/journal.pone.0001481

**Published:** 2008-01-23

**Authors:** M. Abid Hussain, Shaik A. Naveed, Leonardo A. Sechi, Sarita Ranjan, Ayesha Alvi, Irshad Ahmed, Akash Ranjan, Sangita Mukhopadhyay, Niyaz Ahmed

**Affiliations:** 1 Pathogen Evolution Laboratory, Centre for DNA Fingerprinting and Diagnostics (CDFD), Hyderabad, India; 2 Centre for Liver Research and Diagnostics, Deccan College of Medical Sciences and Allied Hospitals, Hyderabad, India; 3 Dipartimento di Scienze Biomediche, Sezione Microbiologia sperimentale e clinica, Facoltà di Medicina, Universiti degli studi de Sassari, Sassari, Italy; 4 Computational and Functional Genomics Group, Centre for DNA Fingerprinting and Diagnostics (CDFD), Hyderabad, India; 5 Laboratory of Molecular and Cellular Biology, Centre for DNA Fingerprinting and Diagnostics (CDFD), Hyderabad, India; University College London, United Kingdom

## Abstract

**Background:**

*H. pylori* causes gastritis and peptic ulcers and is a risk factor for the development of gastric carcinoma. Many of the proteins such as urease, porins, flagellins and toxins such as lipo-polysaccharides have been identified as potential virulence factors which induce proinflammatory reaction. We report immunogenic potentials of isocitrate dehydrogenase (ICD), an important house keeping protein of *H. pylori*.

**Methodology/Principal Findings:**

Amino acid sequences of *H. pylori* ICD were subjected to *in silico* analysis for regions with predictably high antigenic indexes. Also, computational modelling of the *H. pylori* ICD as juxtaposed to the *E. coli* ICD was carried out to determine levels of structure similarity and the availability of surface exposed motifs, if any. The *icd* gene was cloned, expressed and purified to a very high homogeneity. Humoral response directed against *H. pylori* ICD was detected through an enzyme linked immunosorbent assay (ELISA) in 82 human subjects comprising of 58 patients with *H. pylori* associated gastritis or ulcer disease and 24 asymptomatic healthy controls. The *H. pylori* ICD elicited potentially high humoral immune response and revealed high antibody titers in sera corresponding to endoscopically-confirmed gastritis and ulcer disease subjects. However, urea-breath-test negative healthy control samples and asymptomatic control samples did not reveal any detectable immune responses. The ELISA for proinflammatory cytokine IL-8 did not exhibit any significant proinflammatory activity of ICD.

**Conclusions/Significance:**

ICD of *H. pylori* is an immunogen which interacts with the host immune system subsequent to a possible autolytic-release and thereby significantly elicits humoral responses in individuals with invasive *H. pylori* infection. However, ICD could not significantly stimulate IL8 induction in a cultured macrophage cell line (THP1) and therefore, may not be a notable proinflammatory agent.

## Introduction

*Helicobacter pylori* is a Gram negative, curved bacterium that establishes chronic infections in human stomach. It is an important pathogen that is thought to have co-evolved with humans [1], [2]. It inhabits about half of the world population and is considered as a potential risk factor for the development of gastric adenocarcinoma [3]. *H. pylori* is a recognized cause of chronic gastritis, peptic ulcer disease, gastric carcinoma, and mucosa-associated lymphoid tissue (MALT) lymphoma. Several mechanisms have been proposed to explain its role in pathogenesis of the gut. Despite high colonization rates only a small subset of infected people experience *H. pylori*-associated diseases [2]. Associations of *H. pylori* with disease-specific factors have remained more or less enigmatic even though the genome sequences were deciphered almost a decade ago [2].

One of the most distinctive features of *H. pylori* is the genetic diversity between clinical isolates obtained from different patient populations [3]. Recasting of the genome is the norm with this bacterium, creating thereby a lot of allelic and phase variation [3], posing difficulties in the development of diagnostics and vaccines.

Once acquired, the infection persists for years and, elicits detectable immune responses in infected persons [4], [5] characterized by increased levels of specific IgG and IgA in serum and augmentation of secretory IgA and IgM in stomach [6]. Therefore, noninvasive serological tests were recommended and developed for the diagnosis of *H. pylori* infection. Among these, the enzyme-linked immunosorbent assay (ELISA) is one of the most extensively used method as it is relatively affordable, fast, simple to perform, and could be easily adopted for screening large number of samples [7]. Exploring for anti *H. pylori* antibodies is often prescribed before endoscopy or before initiating treatment. Although due to immunological memory, serodiagnosis may not be preferred to check the success or outcome of eradication treatment, it remains a choice as a sensitive method probably because of its non-invasive nature [8].

Currently, there has not been any single ‘gold standard’ antigen, which can unambiguously be used across all populations. One of the important reasons for this could be the extraordinary diversity present in *H. pylori* [9]–[13] and also its specific adaptation in different hosts [12], [13]. Although proteins such as catalase, *gro*EL, and flagellin are strongly immunogenic, they give nonspecific cross-reactions with other Gram-negative bacteria and, more particularly, *Campylobacter jejuni* [14], [15]. In contrast, virulence linked antigens such as CagA or VacA, show quite a high degree of sequence variability, as there exist *H. pylori* strains, which are genetically diverse and phenotypically variable for one or both the antigens [16]–[18]. Despite this, satisfactory associations between serum antibodies and invasive disease have been described [19].

Housekeeping genes of *H. pylori* have been used recently to track population migrations and were found to be stable in the genome across centuries [20]. Housekeeping proteins however, have not been tested by and large for their use in diagnostics or vaccines. The metabolic enzymes seem to be a good candidate to be included into an immuno-diagnostic test kit since they often do not cross-react with sera from healthy controls [21]. Also, owing to their housekeeping function, they are highly conserved among different *H. pylori* strains irrespective of their genotypes and thus can serve as ideal immuno-diagnostic candidates.

Proteins that are released from bacteria during late logarithmic growth phase, such as superoxide dismutase and isocitrate dehydrogenase (ICD), are described as autolysis markers [22]–[24] and some of them have been proved to be excellent diagnostic antigens. For example, ICD of *M. tuberculosis* has been described as a potential antigen to discriminate pulmonary tuberculosis patients from healthy controls [24]. However, previous comparisons of the antigenic patterns of *H. pylori* proteins (including some of the housekeeping proteins) recognized by patient sera revealed no association of specific *H. pylori* antigens with cases of particular gastroduodenal pathology [21].

This study demonstrates interaction of the host immune system with *H. pylori* ICD in the form of significant humoral responses seen in disease subjects. Further, we describe *H. pylori* ICD antigen to distinguish ulcer disease and gastritis patients from uninfected healthy individuals.

## Results

### In silico modelling, expression and purification of H. pylori ICD protein

**Figure 1:**
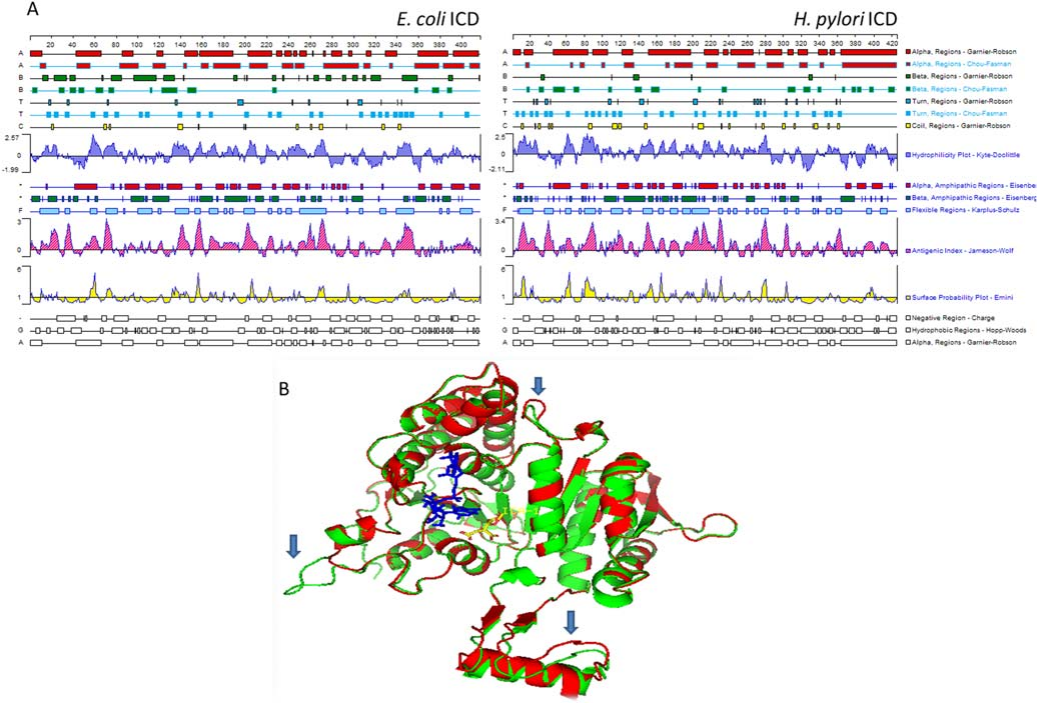
Computational analyses and homology modelling of the *H. pylori* and *E. coli* ICD protein sequences, structures and properties. A. The two proteins were analyzed for different helices, coils and turns, hydrophilicity, antigenicity and surface probability etc. using Protean software in the DNAstar package. Antigenicity index (Jameson-Wolf) of *H. pylori* ICD was found to be higher (3.4) than the *E. coli* ICD. B. We built a homology model of the *H. pylori* ICD (Using Modeller 6) based on crystal structure of *E. coli* ICD (PDB code: 1AI2). Shown here is a comparison of backbone organization of *H. Pylori* (26695) ICD with that of *E. coli*; *E. coli* (red), *H. pylori* (green), NADP (blue), ICA (yellow). Structures of the two ICDs were found to be significantly different as regards to the surface exposed loops (RMS Deviation 0.45) corresponding to putative, immunogenic epitopes (arrows).

Protein sequence of ICD of *H. pylori* was analyzed to predict its immunogenicity based on antigenic index, hydrophilicity and surface probability. Figure 1A shows *in silico* antigenic profiles of ICD generated using Protean software (DNAStar Inc. USA). Briefly, several traces corresponding to immunoreactive amino acid motifs with predictably higher antigenic indices (∼3.4) were identified. The *H. pylori* ICD thus displayed major antigenic stretches (Figure 1A) prompting us to look at it as a putatively immunogenic protein. The antigenicity indices of ICD were comparable to those previously reported for a PPE antigen Rv2430c of mycobacterial origin, which was further proved to be an immunodominant antigen [25]. The *E. coli* ICD was also analyzed in a similar manner and appeared to be putatively immunogenic; there was a clear difference found as compared to *H. pylori* ICD, with respect to the abundance and position of antigenic residues. Later, we constructed a homology model of the *H. pylori* ICD based on the crystal structure of *E. coli* ICD (PDB code 1AI2) and were able to portray backbone organization of *H. Pylori* (26695) ICD along with that of *E. coli*. The two ICDs were found to be significantly similar (RMS Deviation 0.45) yet differed with respect to 3 important surface exposed loop regions which are likely to constitute the areas of structural differences corresponding to the putative antigenic epitopes, possibly imparting differential antibody responses (Figure 1B).

**Figure 2:**
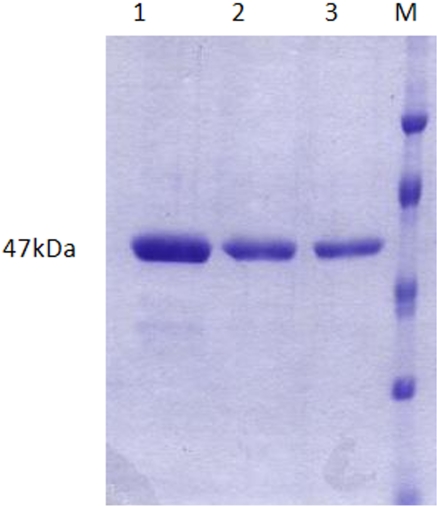
Affinity purification of recombinant ICD of *H. pylori*. His-tagged recombinant protein was purified under native conditions by nickel column chromatography and stained with Coomassie blue after 10% SDS/PAGE. Lanes 1, 2 and 3: *H. pylori* ICD; lane 4: (M) protein molecular mass marker with distribution 35, 45, 66.2 and 116 kDa (beginning bottom).

The over-expressed N-terminal His-tagged ICD was purified to >95% homogeneity on a nickel affinity column (Figure 2). The molecular mass of the recombinant ICD was determined to be 47 kDa. Protein purification was carried out under native conditions with a yield of 2.0 mg of protein per 500 ml of start culture.

### Interaction of H. pylori ICD with the human immune system

**Figure 3:**
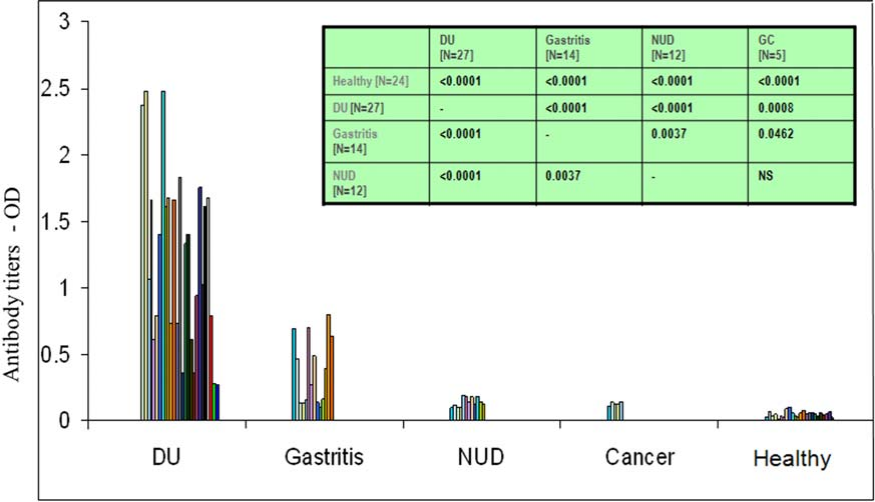
Specific antibody responses to ICD protein depicted as ELISA titers. Serum Antibodies were detected by the recombinant, *H. pylori* ICD in sera samples of patients with gastroduodenal pathology such as gastritis and ulcer and were compared to clinically healthy individuals, 18 of those were detected negative by UBA. Table in the inset reveals significance or otherwise of the student's *t* test for the antibody titers obtained for various disease conditions. Two tailed *p* values were obtained through student's *t* test with a 95% power (level of significance) and a minimally significant *p* value of 0.05.

**Figure 4:**
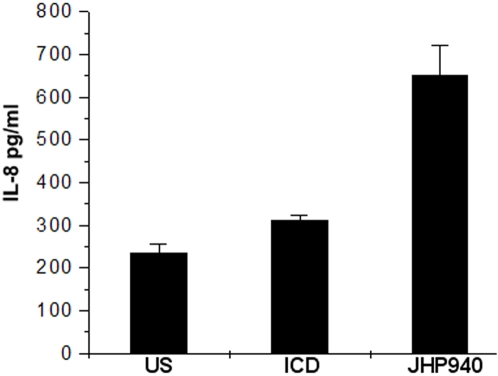
Results of the IL-8 ELISA performed using cultured human macrophages stimulated by the recombinant ICD of *H. pylori*. ELISA titers from unstimulated (US) macrophages were used as negative control and those from macrophages stimulated with a known proinflammatory protein JHP940 [30] were used as a positive control. ICD did not induce any significant IL-8 response as compared to control; the JHP940 protein on the other hand revealed significantly high levels of IL-8 as compared to ICD (*p*<0.0218).

Humoral immune responses directed against the *H. pylori* ICD were compared among patients with *H. pylori* infection (gastritis, NUD, DU and GC) and healthy controls (Figure 3). The immunoreactivity data were statistically analyzed and compared with connection to both infected and healthy sera. These data demonstrated that sera of all of the infected patients belonging to gastritis and duodenal ulcer (DU) categories carried statistically significant (*P*<0.0001) anti ICD antibody levels as compared to those of the healthy controls. The ICD protein, which has an apparently important metabolic role, was thus found to be able to elicit a strong humoral response in subjects with gastritis and DU. However, in cases of non-ulcer dyspepsia (NUD) and gastric cancer (GC), reduced humoral response was seen. Given these observations, it is likely that the release of ICD protein is associated with active, *H. pylori* infection. In contrast to observed humoral responses, the recombinant, *H. pylori* ICD did not induce any significant cellular immune responses as evidenced from lack of secretion of IL-8 from human macrophages induced with recombinant, *H. pylori* ICD *in vitro* (Figure 4). We therefore, suggest that ICD excites only the humoral compartment and may not contribute to gastric pathology or cytokine induced changes in gastric physiology.

## Discussion

Identification and characterization of highly immunogenic *H. pylori* proteins is a compulsory requirement for the development of serological tests based on recombinant purified antigens. Combined with the urea breath test, serology is a noninvasive approach to detect *H. pylori* infection, and several serological test kits based on many different *H. pylori* antigens are commercially available. Although the humoral immune response to *H. pylori* seems to be quite variable, combinations of frequently recognized antigens could prove useful for diagnostic purposes [21]. The metabolic enzymes such as ICD seem to be good candidates to be harnessed for serological test kit development mainly because they do not cross-react with sera from non-infected or healthy individuals [24].

The significance of many immunogenic proteins of *H. pylori* including some metabolic enzymes has not been appreciated much in clinical samples in a previous proteomics based study [21]. Our study was however directed to evaluate purified, recombinant, *H. pylori* ICD for its immunological properties in different classes of *H. pylori* infected acid peptic disease patients. We attempted to systematically examine the presence and usefulness of immune responses elicited by the ICD protein in *H. pylori* infected patients.

Our analysis using recombinant ICD of *H. pylori* revealed significantly high antibody titers among peptic ulcer disease and gastritis patients, as compared with NUD and cancer patients (Figure 3). We speculate that the immune response seen in peptic ulcer cases and gastritis could perhaps be due to high loads of bacteria associated with invasive pathology and thereby a higher number of autolysed pathogens or pathogens presenting ICD on the surface. This may possibly explain the comparatively high-antibody response in invasive category patients.

*H. pylori* antigens are described to be released into the extracellular space via multiple mechanisms such as specific secretion pathways, autolysis, and formation of membrane vesicles [23] and the surface properties of *H. pylori* have been suggested to be unique in allowing adsorption/surface localization of such proteins [26]. ICDs are traditionally known as markers of autolysis [22], [24], [27], released during the late logarithmic phase. The extracellular release of proteins could potentially be occuring only during *in vitro* growth of *H. pylori*. However, *in vivo* existence of such mechanisms, if demonstrated, might be able to explain the role of various secreted proteins in molecular pathology of ulcer disease. The entry of *H. pylori* proteins into the gastric submucosal space may have important functional consequences such as promotion of proinflammatory and chemotactic responses through release of cytokines from activated macrophages. Also, the extracellular release and or adsorption or surface localization [26] of potentially immunogenic proteins might support a bacterial strategy for diverting an effective local immune response [23]. Keeping these multiple mechanisms in mind, we checked proinflammatory potentials of ICD with the help of an IL-8 ELISA assay. In our observation, ICD did not significantly induce IL-8 secretion in differentiated, cultured macrophages. However, since we did not analyze role of ICD as regards to induction of other proinflammatory cytokines such as TNF-α and IL1-β, it will be premature to justly rule out its involvement in gastric inflammation.

Having shown that *H. pylori* ICD elicits a humoral response, we investigated the basis of immune specificity of this protein as reflected by structure based modelling. Our sequence analyses and molecular modelling of *H. pylori* and *E. coli* ICDs did not reveal complete structural overlaps between the two (Figure 1); several regions of structural dissimilarities were discerned which possibly correspond to the putative antigenic epitopes in *H. pylori* ICD. Apart from the issues linked to structural conservation or diversity, we hypothesize that higher reactivity of *H. pylori* ICD as compared to ICD of (commensal) *E. coli* with the host immune system *in vivo* could be associated with 1) different invasiveness of *H. pylori* and *E. coli* 2) different magnitudes of bacterial load, turnover and autolysis 3) different repertoire of antigen presenting cells (macrophages versus dendritic cells) involved and their relative abundance 4) availability of surface exposed epitopes in *H. pylori* ICD (as those highlighted in Figure 1B), and 5) presence of active lesions of chronic inflammation, mucosal damage and chemotactic effects of the other *H. pylori* proteins in the *milieu*.

While considering possible cross-reactivity from other invasive bacteria such as *C. jejuni*, we tested *H. pylori* ICD against 8 samples of *C. jejuni* enteritis patient sera and found that it did not cross-react significantly (data not shown). The *H. pylori* free status of our *C. jejuni* positive patients was determined based on the absence of symptoms of indigestion, stomach pain and heartburn etc. However, since we used only limited sera samples from *C. jejuni* patients, we are not inclined to brand *H. pylori* ICD as a standalone diagnostic antigen. Nonetheless, *C. jejuni* may not always comprise a much competitive background unless *C. jejuni* patients themselves are clinically colonized by *H. pylori* or vice-versa.

Finally, the promising results we obtained in terms of significant humoral responses to *H. pylori* ICD may not be construed as immediately applicable for field level diagnosis. Nonetheless, it is possible that our findings provide a solid foundation for the development of a sero-diagnostic test for *H. pylori* associated gastroduodenal pathologies. We suggest the potential of this antigen and its epitope specific peptides may further be analyzed by using a large battery of sera samples representing different disease subjects. Similarly, the status and significance of cross-reacting antibodies in sera corresponding to illnesses caused by bacteria such as Salmonella spp., Shigella spp. and other invasive enteropathogens might also be determined. It will be worthwhile to explore in-depth the involvement of ICD in signaling activities in the submucosal space, specially its interaction with the activated macrophages and lymphocytes. Also, given its important role in the tricarboxylic acid cycle and the absence of glyoxylate shunt in *H. pylori* [27], the evaluation of ICD as a possible interventional target poses an exciting proposal.

## Materials and Methods

### In silico antigenicity of predicted H. pylori ICD (HP0023)

Nucleotide sequence of *H. pylori icd* (ORF HP0023) and the corresponding amino acid sequence was obtained from Pylorigene database (http://genolist.pasteur.fr) and was subjected to analysis by DNAStar package (DNAStar Inc. Madison, USA). Amino acid sequence of ICD was subjected to scrutiny for any antigenic determinants using Protean program within the DNAStar package. Parameters such as hydrophilicity, surface probability and antigenic index were calculated for the entire protein using this software. Antigenic indices showing more than 2 peaks at >3.0 scales were considered as significantly more antigenic.

### Computational modelling of ICD structure

Using Modeller-6, a molecular modelling program that implements an automated approach to comparative protein structure modelling by satisfaction of spatial restraints [28] was used for determining the three dimensional model of *H. pylori* ICD. The ICD model of *H. pylori* was generated based on the *E. coli* ICD template 1A12 which shares overall 68% sequence identity with the *H. pylori* ICD.

### Production of recombinant H. pylori ICD

The ORF corresponding to *H. pylori icd* (HP0023, 1.275 kb) was amplified by PCR from the genomic DNA of a *H. pylori* patient isolate MS8. The recombinant ICD protein was over expressed in *E. coli* and purified to homogeneity (Figure 2) according to the methods previously described by Banerjee *et al* [24]. The harvest of purified protein was standardized up to 2.0 mg of purified protein per 500 ml of starting culture of recombinant *E. coli*. The purified recombinant protein was dialyzed against 20 mM Tris HCl, pH 7.5, with 100 mM NaCl and 3% glycerol. Further, the purified protein was treated with polymixin B to rule out effects of any LPS contamination. The purified recombinant ICD was lyophilized and immediately stored frozen for downstream use in clinical diagnosis.

### Patients and human serum samples

The study population (*n* = 82) comprised 58 *H. pylori* infected patient samples (for details of number of cases analyzed in each category, please see Figure 3) obtained from cases reporting mainly to the Deccan College of Medical Sciences and Allied Hospitals, (DCMS), Hyderabad, and 24 clinically healthy donors. The healthy control group was comprised of 18 proven *H. pylori* negative cases tested with Urea Breath Assay (UBA) and 6 asymptomatic individuals who were confirmed *H. pylori* negative as tested by commercially available ELISA kit (Pyloriset EIA-GIII, Orion, Espoo, Finland). In all the patient groups, diagnosis of ulcer or gastritis was confirmed or ruled out by endoscopy by the attending gastroenterologist. The presence of *H. pylori* in diseased subjects included in this study was previously confirmed by Gram's staining, a rapid urease test (RUT), 16S rRNA and *cag*A gene based PCRs and culture [29]. The study population had no sex or age bias. Written informed consents were obtained from all the patients and healthy controls at the respective centres.

### ELISA for humoral responses

ELISA assay was performed to check the humoral immune response in humans against recombinant ICD. Briefly, the 96-well microtiter plates were coated with ∼500 ng of recombinant ICD protein. The coated plates were incubated overnight at 4°C, washed three times with PBS-T (0.05% Tween 20 in 1× PBS) and twice with PBS. Plates were then blocked with 100 µl of blocking buffer (2% BSA in 1× PBS) at 37°C for 2 hrs. The plates were washed three times with wash buffer PBS-T and twice with PBS. The *H. pylori*-infected human sera belonging to different clinical groups were serially diluted (1∶50, 1∶100, 1∶200 and 1∶400) in blocking buffer (1% BSA in PBS), to obtain optimum titers. Diluted sera [50 µl of 1∶200 diluted sera] were added to antigen-coated wells and incubated for 1 hr at 37°C. The plates were rinsed thrice with PBS-T and twice with PBS and further incubated with anti-human IgG-horseradish peroxidase (Sigma) at 37°C for 1 hr. Horseradish peroxidase activity was detected using a chromogenic substance, *o*-phenylenediamine tetrahydrochloride (Sigma) in citrate-phosphate buffer (pH 5.4) and H_2_O_2_ as 1 µl/ml. The reactions were stopped by using 0.5 M H_2_SO_4_, and the absorbance values were measured at 492 nm in an ELISA reader (Bio-Rad). Each ELISA was repeated at least twice with all clinical sera isolates. Student's *t* test was performed to calculate *p* values based on the means of the serum antibody titers corresponding to healthy and infected classes by using the online scientific calculator of GraphPad (www.graphpad.com_quickcalcs_ttest1.cfm). *P* values less than 0.05 were considered statistically significant.

### ELISA for proinflammatory cytokine IL-8

THP-1 cells (human Monocyte, ATCC TIB 202) were grown and maintained in RPMI 1640 (Invitrogen) supplemented with 10% fetal bovine serum, 2 mM Glutamine and 1% anti-bacterial and anti-mycotic solution (Gibco). For macrophage like stage, approximately 1×10^−6^ cells/well were differentiated using 5 ng/ml Phorbol-12 myristate 13c acetate (PMA, Sigma). After 24 hours, cells were washed once with RPMI 1640 media and stimulated using 10 µg/ml of recombinant ICD. A proinflammatory protein, HP940, of *H. pylori* [30], purified under similar conditions was used as a positive control (1 ug/ml). Cells were incubated for 24 hours in a humidified atmosphere at 37°C. Cells without protein treatment (unstimulated cells) served as negative control. Culture supernatant collected after 24 hours was used for the estimation of IL-8 using commercially available optEIA ELISA Kit (BD Biosciences). Assay was performed according to the instruction of manufacturer and the cytokine levels were calculated using the recombinant standard provided in the kit. Sensitivity of IL-8 was 3.1 pg/ml. Values were expressed as mean± SD.
